# Frequency and impact of confounding by indication and healthy vaccinee bias in observational studies assessing influenza vaccine effectiveness: a systematic review

**DOI:** 10.1186/s12879-015-1154-y

**Published:** 2015-10-17

**Authors:** Cornelius Remschmidt, Ole Wichmann, Thomas Harder

**Affiliations:** Immunization Unit, Robert Koch Institute, Seestrasse 10, 13353 Berlin, Germany

**Keywords:** Influenza, Vaccination, Bias, Confounding by indication, Healthy user bias, Effectiveness

## Abstract

**Background:**

Evidence on influenza vaccine effectiveness (VE) is commonly derived from observational studies. However, these studies are prone to confounding by indication and healthy vaccinee bias. We aimed to systematically investigate these two forms of confounding/bias.

**Methods:**

Systematic review of observational studies reporting influenza VE and indicators for bias and confounding. We assessed risk of confounding by indication and healthy vaccinee bias for each study and calculated ratios of odds ratios (crude/adjusted) to quantify the effect of confounder adjustment. VE-estimates during and outside influenza seasons were compared to assess residual confounding by healthy vaccinee effects.

**Results:**

We identified 23 studies reporting on 11 outcomes. Of these, 19 (83 %) showed high risk of bias: Fourteen due to confounding by indication, two for healthy vaccinee bias, and three studies showed both forms of confounding/bias. Adjustment for confounders increased VE on average by 12 % (95 % CI: 7–17 %; all-cause mortality), 9 % (95 % CI: 4–14 %; all-cause hospitalization) and 7 % (95 % CI: 4–10 %; influenza-like illness). Despite adjustment, nine studies showed residual confounding as indicated by significant off-season VE-estimates. These were observed for five outcomes, but more frequently for all-cause mortality as compared to other outcomes (*p* = 0.03) and in studies which indicated healthy vaccinee bias at baseline (*p* = 0.01).

**Conclusions:**

Both confounding by indication and healthy vaccinee bias are likely to operate simultaneously in observational studies on influenza VE. Although adjustment can correct for confounding by indication to some extent, the resulting estimates are still prone to healthy vaccinee bias, at least as long as unspecific outcomes like all-cause mortality are used. Therefore, cohort studies using administrative data bases with unspecific outcomes should no longer be used to measure the effects of influenza vaccination.

**Electronic supplementary material:**

The online version of this article (doi:10.1186/s12879-015-1154-y) contains supplementary material, which is available to authorized users.

## Background

Since randomized controlled trials (RCTs) assessing the effects of influenza vaccination on clinical outcomes are scarce, evidence on influenza vaccine effectiveness (VE) mainly derives from observational studies [1]. However, these studies are prone to bias and have been suspected to systematically overestimate VE, particularly against unspecific outcomes such as all-cause mortality and among the elderly [2]. Although it has been accepted that observational studies are susceptible to bias, there is an ongoing controversy whether and to what extend confounding by indication and healthy vaccinee bias affect influenza VE estimates [3–9]. Both forms of bias/confounding have been described in such studies, but it is important to note that their presence has opposing consequences for the VE estimates: “confounding by indication” is likely to be present if patients with underlying chronic diseases are more likely to be vaccinated than healthy study participant. If no adequate statistical adjustment (e.g., for comorbidities) is made, this leads to an underestimation of VE since the less healthy population is at higher risk of adverse health outcomes. The alternative scenario is called “healthy vaccinee bias” and refers to a situation when patients, who are in better health conditions, are more likely to adhere to the annually recommended influenza vaccination [10]. If not corrected for (e.g., by adjustment for comorbidities or indicators of health seeking behavior), healthy vaccinee bias leads to an overestimation of VE.

To test whether residual confounding by healthy vaccinee effects is still present in the adjusted data, it has been suggested by some authors that investigators should obtain “off-season” estimates. Off-season estimates are calculated for time periods outside influenza seasons when the virus is (virtually) not circulating and therefore no vaccine effect should be present [10, 11]. Any VE obtained during this control period would be attributable to unmeasured confounding, whereas successful adjustment would have removed the effect.

A systematic analysis of these two forms of bias/confounding and their consequences for influenza VE studies has not been published so far. We therefore addressed this issue by a systematic review.

## Methods

### Question framing

This study addressed the following questions: (i) How often do observational studies on influenza VE show indication of confounding by indication and/or healthy vaccinee bias? (ii) What is the impact on VE point estimates? And (iii) how many of these studies show indication of unmeasured (residual) confounding in the adjusted analyses? To define the conceptual framework of the study, we identified five indicators from the literature, which allow conclusions on the presence of the two forms of bias/confounding in the included studies (Table 1).

**Table 1:** Conceptual framework: Indicators and conclusions for presence of confounding by indication and healthy vaccinee bias in influenza vaccine effectiveness studies

Indicator	Conclusion	References
Vaccinated study participants have a higher proportion of comorbidities than unvaccinated study participants, as indicated by baseline characteristics	High risk of confounding by indication in the unadjusted data set	[6, 38]
Vaccinated study participants have a lower proportion of comorbidities than unvaccinated study participants, as indicated by baseline characteristics	High risk of healthy vaccinee bias in the unadjusted data set	[35, 36]
Inclusion of comorbidities in the regression model increases vaccine effectiveness	Confounding by indication has led to underestimation of vaccine effectiveness in the unadjusted data set	[7]
Inclusion of comorbidities in the regression model decreases vaccine effectiveness	Healthy vaccinee bias has led to overestimation of vaccine effectiveness in the unadjusted data set	[7]
Significant effects of influenza vaccination appear outside the influenza season (“off-season estimates”), despite adjustment for comorbidities	Residual confounding by healthy vaccinee bias	[3, 11, 36]

### Study protocol

We performed the systematic review according to the Preferred Reporting Items for Systematic Reviews and Meta-analyses (PRISMA) statement [12]. The respective protocol for this review is shown in Additional file 1.

### Eligibility criteria

Studies were included if they fulfilled the following criteria defined a priori: (i) observational (non-randomized) study; (ii) calculated influenza VE by comparing vaccinated and unvaccinated participants; (iii) reported baseline characteristics of vaccinated and unvaccinated participants; (iv) reported data on at least one clinical outcome; (v) reported crude and confounder-adjusted VE estimates from at least one influenza season; (vi) reported confounder-adjusted VE estimates from at least one “control” period outside the influenza season (off-season estimate).

### Literature search

Two reviewers (CR and TH) searched MEDLINE, EMBASE and Cochrane Central Register of Controlled Trials (date of last search: 25.05.2014) and independently screened each citation and subsequent full text articles. The complete search strategy is shown in Additional file 2. Electronic searches were complemented by manually searching the reference lists of all identified studies and reviews for additional studies. No restrictions were made regarding publication language and publication status (published/unpublished).

### Data extraction

From each included study, two investigators (CR and TH) independently extracted the following information: country, study design, age, sex, characteristics of study population (e.g., patients with underlying comorbidities), source of patient data, identification of clinical outcomes and vaccination status, definition of influenza season and off-season, and population size. In addition, we extracted data on crude and adjusted VE point estimates for all reported outcomes during influenza seasons, adjusted off-season point estimates, and which confounder were considered. Extraction forms were pilot tested with the first two identified studies.

### Assessment of risk of bias

Two investigators (CR and TH) independently assessed risk of bias. In case of disagreements, a final decision was made by consensus or resolved by a third reviewer (OW). We used the predefined criteria derived from the above mentioned methodological framework (see Table 1) to assess the risk of healthy vaccinee bias and confounding by indication in the included studies: A study was judged to be at high risk of healthy vaccinee bias if vaccinated participants had significantly fewer comorbidities (or respective indicators such as medical visits) than unvaccinated participants, as indicated by baseline characteristics. A study was judged to be at high risk of confounding by indication if vaccinated participants had significantly more comorbidities (or respective indicators) than unvaccinated participants, as indicated by baseline characteristics. For case–control studies, vaccinated and unvaccinated participants of the control groups were compared. The results of these assessments were expressed as a considered judgment, using the categories “high risk of bias”, “low risk of bias” and “unclear risk of bias”.

Two approaches, a descriptive and a meta-analytical, were used to assess whether the included studies successfully corrected for bias/confounding. First, we compared crude VE estimates to confounder-adjusted in-season estimates from the same studies. If the studies reported more than one confounder-adjusted estimate, we used the fully adjusted model. If adjustment increased the estimated VE, we concluded that data were at least in part corrected for confounding by indication. If adjustment decreased the estimated VE, we concluded that data were at least in part corrected for healthy vaccinee bias (see Table 1).

Second, we used the approach suggested by Hrobjartsson et al. [13, 14] to quantify the extent by which adjustment for confounders increased the in-season estimate compared to the crude estimate: For each outcome for which more than one study reported data, we calculated the ratio of odds ratios (crude/adjusted VE during influenza season) per study. A ratio of >1 indicates that adjustment led to a stronger effect of vaccination, i.e., an increased VE. For calculation of 95 % CI, we used the formula provided by Hrobjartsson et al. [13]. To quantify the impact of adjustment for confounders, we then meta-analysed the individual study ratios of odds ratios for each outcome separately, using random-effects models with inverse-variance methods. For this analysis, we excluded two studies [15, 16] which did not report 95 % CI for the respective point estimates.

To evaluate the presence and magnitude of off-season VE estimates, being proposed as indicators of healthy vaccinee bias, we contrasted confounder-adjusted in-season estimates to “pseudo-effectiveness” estimates measured during off-seasons.

All statistical analyses were performed using STATA 12 (StataCorp LP, Texas, USA).

## Results

**Fig. 1:**
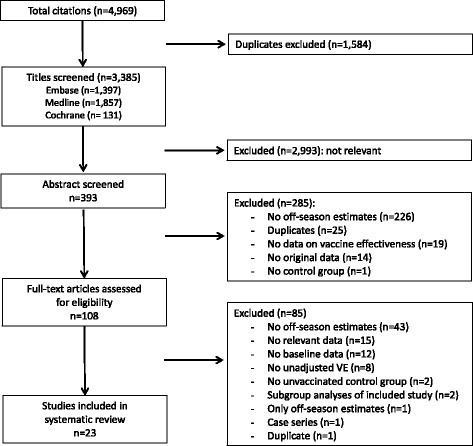
Flow chart for the systematic review

By systematic literature search we identified 3385 publications, of which 23 were finally included in our analysis (Fig. 1) [3, 5, 7, 15–33]. Details on the excluded studies are reported in Additional file 3. Baseline characteristics of the 23 included studies are shown in Table 2. Of these, 20 were cohort studies, while the remaining three had a case–control design. The studies were conducted in North America (*n* = 14), Europe (*n* = 6), Taiwan (*n* = 2) or in multiple continents (*n* = 1) and mainly used disease classification codes (e.g., ICD-9) or civil register data for the identification of outcomes. In three studies interviews were conducted or self-administered questionnaires were applied to collect primary data on relevant outcomes or vaccination status [24, 25, 27]. Except of four studies, which were either performed in students (*n* = 1), in adults aged 40+ years (*n* = 1), or in women who recently experienced live birth (*n* = 2), all studies were conducted in elderly persons. Seven studies covered populations with underlying comorbidities, namely with (chronic) heart disease (CHD), [21, 22] end-stage renal disease (ESRD), [17, 23] chronic obstructive pulmonary disease (COPD), [28, 29] or patients with diabetes or vascular disease [33].

**Table 2:** Baseline characteristics of included studies

Author, year	Country	Study design	Influenza season(s)	Age-group (yrs) or risk group	Age (yrs), range or mean (± SD)	% male	Data sources	Identification of outcomes	Study size (n)
Bond et al., 2012 [17]	US	Cohort	2005/06	Patients with ESRD	V, 60.6 (15.2)	V, 52.5	3 ESRD Networks, records of the US Renal Database (USRDS)	All-cause death through ESRD death notification form	20,220 (without pneumococcal vaccine)
					UV, 57.9 (15.9)	UV, 50.8			
Campitelli et al., 2010 [7]	Canada	Cohort	8 seasons between 1996 and 2007	Elderly ≥ 65	V, 75.3 (6.6)	V, 40.8	National health surveys data linked to Ontario Health Insurance (OHIP) and Discharge Abstract (CIHI) databases	Registered persons database and ICD-9/-10 admission codes	V, 14,512
					UV, 74.2 (6.7)	UV, 40.7			UV, 11,410
Foster et al., 1992 [18]	US	Case–control (matched)	1989/90	Elderly ≥ 65	V, 65-94+	V, 50.8	Databases of participating hospitals	Hospital discharge ICD-9 codes	Cases, 721
					UV, 65-94+	UV, 47.3			Controls, 1786
France et al., 2006 [34]	US	Cohort	1995/96-2000/01	Women and their newborns	V, 30.8 (5.5)	NA	Health maintenance organization (Kaiser Permanante and Group Health Cooperative)	ICD-9 codes for medically attended acute respiratory illnesses in infants	V, 3160
					UV, 29.7 (5.5)				UV, 37,969
Groenwold et al., 2009 [19]	Netherlands	Cohort	1995/96-2002/2003	Elderly ≥ 65	V, 75 (median)	V, 39.4	GPRD of University Medical Center Utrecht	ICPC coding system	V, 37,501
					UV, 74 (median)	UV, 35.2			UV, 13,405
Hottes et al., 2011 [20]	Canada	Cohort	2000/01-2005/06	Elderly ≥ 65	V, 75 (median)	V, 43	Manitoba Immunization Monitoring System (MIMS) and Manitoba health policy database	All-cause mortality or hospital admission ICD-9/-10 codes	139,185 (00/01) to 140,735 (05/06)
					UV, 73 (median)	UV, 44			
Jackson et al., 2006 [35]	US	Cohort	1995/96-2002/3	Elderly ≥ 65	V, 51 % >74	V, 42.7	Health maintenance organization (Group Health Cooperative)	All-cause mortality or hospital ICD-9 discharge codes	72,527
					UV, 46 % >74	UV, 41.9			
Jackson et al., 2002 [21]	US	Cohort	1992-1996	Patients with nonfatal MI	All subjects, 64 (median)	among ≥ 65:	Health maintenance organization (Group Health Cooperative)	Hospital discharge ICD-9 codes, confirmed by chart review	V, 1016
									UV, 362
						V, 47			
						UV, 59			
Jackson et al., 2008 [5]	US	Case–control (matched)	2000/1-2001/2	Elderly 65-94	Cases, 62 % >74	cases, 51	Health maintenance organization (Group Health Cooperative)	ICD-9 code for CA pneumonia and validation using hospital records	Cases, 1173 Controls, 2346 (1838 V, 508 UV)
Johnstone et al., 2012 [33]	40 countries	Cohort	2003/04-2006/7	Elderly ≥ 65 with VD or diabetes	Mean (4 seasons)	Range (4 seasons)	Clinical databases from two RCTs (ONTARGET- and TRANSCEND-trial)	Outcomes adjudicated by independent committee using clinical data	31,546
					V, 67-68	V, 72-73			
					UV, 65-66	UV, 67-70			
Liu et al., 2012 [22]	Taiwan	Cohort	2002-2006	Elderly > 65 with heart disease	V, 74.8 (6.3)	V, 58.3	National Health Research Institute-released cohort dataset	ICD-9 codes for heart diseases	V, 2760
					UV, 75.7 (7.0)	UV, 51.8			UV, 2288
Mangtani et al., 2004 [16]	UK	Cohort	1989/90-1998/99	Elderly ≥ 65	Not reported	Not reported	General practice research database (GPRD)	ICD-9 codes for acute respiratory illnesses; all respiratory-related deaths	Person-years: influenza season, 419,748 summer, 692,415
McGrath et al., 2012 [23]	US	Cohort	1997-1999 and 2001	Patients with ESRD	Mean (4 seasons)	Range (4 seasons)	Medicare claims from the US Renal Data System (USRDS)	All-cause death through ICD-9 codes; Medicare claims from the USRDS	107,465 (1997) to 126,699 (2001)
					V, 62.3-63.9	V, 52.2-53			
					UV, 60.3-61.7	UV, 50.4-51.6			
Nicol et al., 2008 [24]	US	Cohort	2002/03-2005/06	Adults (students)	V, 25.2 (7.9)	V, 25.5	Internet-based survey	Self-reported occurrences of ILI and health care use	12,795
					UV, 23.3 (6.3)	UV, 29.4			
Nicol et al., 2009 [25]	US	Cohort	2006/07	Adults, 50-64	Not reported	V, 24	Internet-based survey	Self-reported occurrences of ILI and health care use	479
						UV, 16			
Ohmit et al., 1995 [26]	US	Case–control (matched)	1990/91-1991/92	Elderly ≥ 65	not reported	not reported	Admission and discharge data of 21 participating hospitals	ICD-9 hospitalization code for pneumonia/influenza	Cases, 1557 Controls, 3401
Omer et al., 2011 [27]	US	Cohort	2004/05-2005/06	Women and their newborns	V, 18.3 % < 19	NA	Georgia Pregnancy Risk Assessment Monitoring System (PRAMS)	PRAMS database (questionnaire or interview)	V, 578
									UV, 3590
					UV, 14.5 % < 19				
Örtqvist et al., 2007 [15]	Sweden	Cohort	1998/99-2000/01	Elderly ≥ 65	V, 51 % >74	V, 41.3	Population register (via national identification number)	ICD-9/-10 codes and cause of death register	260,155
					UV, 51 % >74	UV, 39.0			
Schembri et al., 2009 [28]	UK	Cohort	1998-2006	Adults > 40 with COPD	V, 27 % > 69	V: 42.8	The Health Improvement Network database (THIN), covering data of general practices	Diesease classification codes of THIN databases (“Read codes”)	V, 9679
									UV, 31,062
					UV, 12 % > 69	UV: 42.8			
Sung et al., 2014 [29]	Taiwan	Cohort	2000-2007	Elderly ≥ 55 with COPD	V, 20 % >74	V: 58.7	Reimbursement claims from National Health Insurance Research Database (NHIRD)	ICD-9 codes for acute MI or angina pectoris with invasive therapy	V, 3027
					UV, 17 % >74	UV: 60.8			UV, 4695
Tessmer et al., 2011 [30]	Germany	Cohort	2002-2006	Adults with pneumonia (CAP)	V, 67.6 (14.5) *1	V: 57.2	National Community Acquired Pneumonia Competence Network (CAPNET)	CAPNET database entries	V, 1721
									UV, 3279
					UV, 55.7 (19.0)	UV: 52.5			
Vila-Córcoles et al., 2007 [31]	Spain	Cohort	2002-2005	Elderly ≥ 65	V, 51 % > 74	V, 44.3	Databases of Primary Health Care Centres (PHCC)	ICD-9 codes of PHCC and Civil Registry Offices	V, 6051
									UV, 5189
					UV, 38 % > 74	UV, 43.6			
Wong et al., 2012 [32]	Canada	Cohort	2000/01-2008/09	Elderly ≥ 65	V, 75.5 (6.6)	V, 43.8	Ontario Health administrative databases	ICD-9/ -10 codes of databases and registered persons database	1,297,051 (00/01) to 1,527,364 (08/09)
					UV, 74.5 (6.8)	UV, 43.6			

*SD* standard deviation, *V* vaccinated, *UV* unvaccinated, *ESRD* end-stage renal disease, *ICD* international classification of disease, *CHD* chronic heart disease, *GPRD* general practice research database, *ICPC* international classification of primary care, *MI* myocardial infarction, *CA(P)* community acquired (pneumonia), *VD* vascular disease, *ONTARGET-trial* Ongoing Telmisartan Alone and in Combination With Ramipril Global EndPoint Trial, *TRANSCEND-trial* Telmisartan Randomized Assessment Study in ACE Intolerant Subjects with Cardiovascular Disease, *ILI* influenza-like illness, *COPD* chronic obstructive pulmonary disease

### Reported outcomes

The included studies reported VE estimates (crude and adjusted in-season plus adjusted off-season estimates) related to 11 different clinical outcomes: all-cause mortality (*n* = 12 studies), death due to respiratory event (*n* = 2), major adverse vascular event (*n* = 1), hospitalization due to influenza and/or pneumonia (*n* = 7), hospitalization for acute coronary syndrome (*n* = 1), influenza-like illness (*n* = 3), cardiac death (*n* = 1), hospitalization due to cardiovascular disease (*n* = 1), prematurity (*n* = 1), small for gestational age (*n* = 1), and medically attended respiratory infections in infants (*n* = 1). None of the clinical outcomes was required to be confirmed by laboratory testing for influenza viruses.

### Risk of healthy vaccinee bias and confounding by indication

Of the included 23 studies, 19 (83 %) showed a high risk of bias (either healthy vaccine bias, confounding by indication, or both). Two studies we judged to be at high risk of healthy vaccinee bias but not confounding by indication (Table 3). One of these studies was performed in patients with end-stage renal disease, with vaccinated participants having more favorable prognostic markers than unvaccinated participants; [23] the other study covered patients suffering from COPD and indicated that vaccinated patients had less (severe) comorbidities as indicated e.g., by the Charlson comorbidity index, when compared to unvaccinated patients.

**Table 3:** Risk of healthy vaccinee bias and confounding by indication in the included studies, as judged from the baseline characteristics of vaccinated and unvaccinated participants

Study	Healthy vaccinee bias^a^	Confounding by indication^b^	Indicated by
Bond et al. (2012) [17]			Vaccinated participants have more comorbidities; unvaccinated have worse laboratory values
Campitelli et al. (2011) [7]			Vaccinated participants have more comorbidities; unvaccinated patients have worse functional status
Foster et al. (1992) [18]			More comorbidities in vaccinated participants
France et al. (2006) [34]			More comorbidities in vaccinated participants
Groenwold et al. (2009) [19]			More comorbidities, medications and medical visits in vaccinated participants
Hottes et al. (2011) [20]			More medical visits in vaccinated participants
Jackson et al. (2002) [21]			More comorbidities in vaccinated participants
Jackson et al. (2006) [3]			More comorbidities in vaccinated participants
Jackson et al. (2008) [5]			No major differences in baseline characteristics between groups
Johnstone et al. (2012) [33]			Vaccinated participants have more CAD; unvaccinated have more diabetes and hypertension
Liu et al. (2012) [22]			More comorbidities in vaccinated participants
Mangtani et al. (2004) [16]			More comorbidities and medications in vaccinated participants
McGrath et al. (2012) [23]			Better adherence to dialysis and fewer years with end-stage renal disease in vaccinated participants
Nichol et al. (2008) [24]			More comorbidities in vaccinated participants
Nichol et al. (2009) [25]			No major differences in baseline characteristics between groups
Ohmit et al. (1995) [26]			Unclear data and description
Omer et al. (2011) [27]			Some comorbidities different (diabetes) between groups, other not (hypertension)
Örtqvist et al. (2007) [15]			More comorbidities in vaccinated participants
Schembri et al. (2009) [28]			More comorbidities in vaccinated participants
Sung et al. (2014) [29]			More comorbidities in unvaccinated participants
Tessmer et al. (2011) [30]			More comorbidities in vaccinated participants
Villa-Corcoles et al. (2007) [31]			More comorbidities in vaccinated participants
Wong et al. (2012) [32]			More comorbidities and medications in vaccinated participants

green circle/+: low risk of bias; red circle/-: high risk of bias; yellow circle/?: unclear risk of bias; CAD, coronary artery disease

^a^indicated by: vaccinated participants were healthier (fewer comorbidities) than unvaccinated participants at study entry (cohort studies) or vaccinated controls were healthier (fewer comorbidities) than unvaccinated controls (case–control studies)

^b^indicated by: vaccinated participants were sicker (more comorbidities) than unvaccinated participants at study entry (cohort studies) or vaccinated controls were sicker (more comorbidities) than unvaccinated controls (case–control studies)

Fourteen studies showed a high risk of confounding by indication, but not of healthy vaccinee bias. In 13 of them, [3, 15, 16, 18, 19, 21, 22, 24, 28, 30–32, 34] this was indicated by a significantly higher proportion of vaccinated patients with comorbidities (compared to unvaccinated participants), whereas in one study [20] medical visits served as indicator. In three studies, we found indication for both types of bias/confounding occurring simultaneously [7, 17, 33]. In these studies, the group of vaccinated participants had a higher proportion of comorbidities, while at the same time unvaccinated participants showed a higher proportion of functional impairments or other relevant comorbidities. In a further three studies, [5, 25, 27] no major differences in baseline characteristics between vaccinated and unvaccinated study participants were found. In the remaining one study, risk of bias was unclear due to unclear data and reporting (Table 3) [26].

### Adjustment for confounders and impact on point estimates

In ten of 12 studies reporting on all-cause mortality, adjustment for confounders increased the estimate of VE. The same effect of adjustment was observed in all studies reporting on hospitalization, major adverse vascular events, influenza-like illness and cardiac death. For the remaining outcomes, the effect was either very small or adjustment decreased the VE estimate. All studies adjusted at least for age and comorbidities, although definitions of the latter differed between individual studies (Table 4).

**Table 4:** Crude and confounder-adjusted estimates of vaccine effectiveness during the influenza season in the included studies

Outcome by study	Crude OR (95 % CI)	Adjusted OR (95 % CI)	Confounders considered in the adjusted analysis
All-cause mortality			
Bond et al. (2012) [17]	0.79 (0.72–0.87)	0.73 (0.67–0.81)	Age, race, sex, time on dialysis, diagnostic mode, diabetes, comorbidities, laboratory parameters
Campitelli et al. (2011) [7]	0.65 (0.51–0.84)^a^	0.61 (0.47–0.79)	Demographics, comorbidities, health care utilization, functional status indicators
Groenwold et al. (2009) [19]	0.86 (0.69–1.06)	0.56 (0.45–0.69)	Age, sex, prior healthcare use (GP visits), comorbidities, medication use
Hottes et al. (2011) [20]	0.87 (0.80–0.94)	0.70 (0.64–0.77)	Age, sex, SES, residency, prior influenza/pneumococcal vaccination, medical visits, Elixhauser index
Jackson et al. (2006) [35]	0.56 (0.52–0.61)^b^	0.51 (0.47–0.55)	Age, sex, comorbidities, previous pneumonia hospitalization, number of outpatient visits
Liu et al. (2012) [22]	0.40 (0.34–0.47)	0.42 (0.35–0.49)	Age, comorbidities
McGrath et al. (2012) [23]	0.77 (0.76–0.78)^c^	0.71 (0.70–0.72)^c^	Age, race, sex, cause of ESRD, vintage, adherence, hospital days, mobility aids, comorbidities, oxygen
Örtqvist et al. (2007) [15]	0.50 (−)	0.56 (0.52–0.60)^d^	Age and sex, socioeconomic status, marital status, comorbidities
Schembri et al. (2009) [28]	0.70 (0.58–0.86)^e^	0.59 (0.57–0.61)	Age, sex, year and serious comorbidities
Tessmer et al. (2011) [30]	0.85 (0.61–1.17)	0.63 (0.45–0.89)	Age, sex, pneumococcal vaccination status, body mass index, nursing home residency, smoking, previous antibiotic therapy, long-term oxygen therapy, number of comorbidities
Villa-Corcoles et al. (2007) [31]	0.77 (0.65–0.89)	0.63 (0.54–0.74)	Age, sex, chronic lung disease, chronic heart disease, diabetes, hypertension, immunocompromised, immunocompromised x age
Wong et al. (2012) [32]	0.72 (0.67–0.77)	0.67 (0.62–0.72)	Demographics, comorbidities, use of health care service, medication use, special medical procedures
Death due to respiratory event			
Schembri et al. (2009) [28]	0.3 (0.0–7.4)^e^	0.63 (0.55–0.77)	Age, sex, year and serious comorbidities
Mangtani et al. (2004) [16]	1.32 (−)	0.88 (0.84–0.92)	Risk, age, repeat prescription status
Major adverse vascular event (cardiovascular death or nonfatal myocardial infarction or nonfatal stroke)			
Johnstone et al. (2012) [33]	0.77 (0.61–0.97)^f^	0.65 (0.58–0.74)	Propensity score (body mass index, age, sex, ethnicity, education, vitamin use, smoking history, alcohol use, history of pneumococcal vaccination), history of coronary artery disease, diabetes, hypertension, stroke, admission to nursing home, use of aspirin, ß-blocker, lipid-lowering drug, angiotensin-converting enzyme inhibitor, angiotensin II inhibitor
Hospitalization due to influenza and/or pneumonia			
Foster et al. (1992) [18]	0.78 (−)^g^	0.55 (0.36–0.86)	Sex, race, age, information source, hospital type, region, survival, months, duration of recall
Hottes et al. (2011) [20]	1.09 (0.98–1.21)	0.94 (0.82–1.07)	Age, sex, SES, residency, prior influenza/pneumococcal vaccination, medical visits, Elixhauser index
Jackson et al. (2006) [35]	0.82 (0.75–0.89)^b^	0.71 (0.65–0.78)	Age, sex, comorbidities, previous pneumonia hospitalization, number of outpatient visits
Jackson et al. (2008) [5]	1.04 (0.88–1.22)^b^	0.92 (0.77–1.10)	Age, sex, asthma, smoking, antibiotics, FEV1, oxygen, previous pneumonia, steroids, other drugs
Mangtani et al. (2004) [16]	1.18 (−)^g^	0.79 (0.74–0.83)	Risk, age, repeat prescription status
McGrath et al. (2012) [23]	0.90 (0.87–0.92)^c^	0.84 (0.82–0.86)^c^	Age, race, sex, cause of ESRD, vintage, adherence, hospital days, mobility aids, comorbidities, oxygen
Ohmit et al. (1995) [26]	1.0 (0.82–1.22)^h^	0.68 (0.54–0.86)^h^	Sex, age, smoking, information source, region, survival, hospital type
Hospitalization for acute coronary syndrome			
Sung et al. (2014) [29]	0.52 (0.41–0.66)	0.45 (0.35–0.57)	Age, gender, comorbidity condition, hypertension, diabetes, dyslipidemia, arrhythmia, anemia, pneumonia, monthly income, level of urbanization, geographic region
Influenza-like illness			
McGrath et al. (2012) [23]	0.93 (0.91–0.95)^c^	0.88 (0.86–0.89)^c^	Age, race, sex, cause of ESRD, vintage, adherence, hospital days, mobility aids, comorbidities, oxygen
Nichol et al. (2008) [24]	0.77 (−)^g^	0.70 (0.56–0.89)	Age, sex, high-risk status, smoking, general health, undergraduate status, medical visits, virus match
Nichol et al. (2009) [25]	0.55 (−)^g^	0.48 (0.27–0.86)	Sex, smoking, general health, high-risk status, functionality, activity limits, previous vaccination
Cardiac death^i^			
Jackson et al. (2002) [21]	1.24 (0.84–1.84)^j^	1.06 (0.63–1.78)	Age, gender, severe heart failure during hospitalization, smoking status, comorbidities, medication
CVD hospitalization			
Liu et al. (2012) [22]	0.85 (0.76–0.94)	0.84 (0.76–0.93)	Age, comorbidities
Prematurity			
Omer et al. (2011) [27]	0.56 (0.33–0.96)^k^	0.40 (0.24–0.68)^k^	Gestational age, maternal age, multiple births, maternal risk factors and comorbidities, labor/delivery complications, birth defects, insurance, smoking, alcohol, race, education, marital status, weight
Small for gestational age			
Omer et al. (2011) [27]	0.73 (0.40–1.33)^k^	0.68 (0.32–1.46)^k^	Gestational age, maternal age, multiple births, maternal risk factors and comorbidities, labor/delivery complications, birth defects, insurance, smoking, alcohol, race, education, marital status, weight
Medically attended respiratory illness in infants			
France et al. (2006) [34]	0.90 (0.80–1.02)^l^	0.96 (0.87–1.07)^l^	Infant gestational age at birth, infant sex, maternal age, Medicaid coverage, maternal history of prior influenza vaccination, and maternal high-risk status

^a^adjusted for season and demographics; ^b^adjusted for age and sex; ^c^data were pooled first from 4 seasons; ^d^adjusted point estimates from season 98/99; ^e^unadjusted odds ratios and 95 % CI calculated from death rates for all seasons (1988–2006); ^f^data were pooled first from 4 seasons; ^g^95 % CI not reported; ^h^data from season 1990/91 were used; ^i^defined as death due to myocardial infarction, ischemic heart disease, congestive heart failure, hypertensive heart disease, cardiac arrest, and atrial fibrillation; ^j^adjusted for age; ^k^point estimates reported here were calculated for local influenza activity and included periods of regional and widespread influenza activity; ^l^matched by study site and birth week

**Fig 2:**
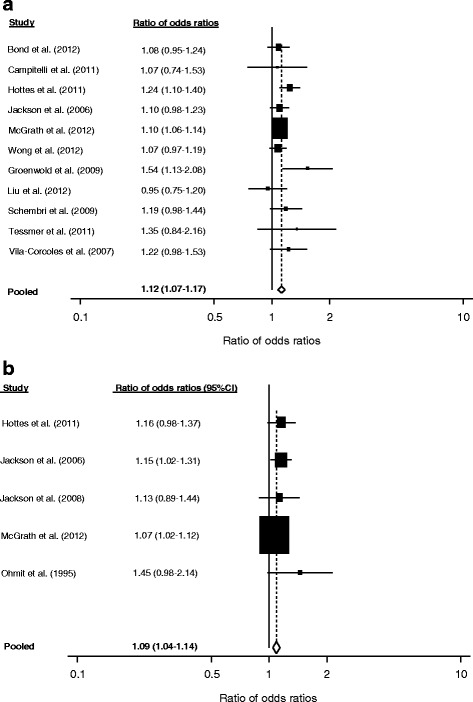
Impact of adjustment for confounders, expressed as ratio of odds ratios (crude/adjusted): (**a**) All-cause mortality, (**b**) Hospitalization due to influenza or pneumonia

We pooled the data for the outcomes all-cause mortality, hospitalization due to influenza or pneumonia, and ILI since more than one study reported on these outcomes. For all-cause mortality, this ratio of odds-ratio analysis indicated that adjustment for confounders increased the effect of vaccination by 12 % (95 % CI: 7–17 %) (Fig. 2a). For hospitalization due to influenza or pneumonia, effect size increased by 9 % (95 % CI: 4–14 %) after adjustment for confounders (Fig. 2b). For the outcome ILI, adjustment for confounders increased VE estimate by 7 % (95 % CI: 4–10 %).

### Off-season estimates

The included 23 studies reported a total 31 off-season estimates. Three of the studies reported pre-season as well as post-season estimates [7, 20, 35]. Two studies reported only pre-season estimates, [5, 23] while five studies provided data on post-seasons “effectiveness” only [15, 16, 18, 19, 32]. The remaining studies reported off-season estimates either for the whole period outside the influenza season or for single months before and after the seasons. Most studies defined beginning and end of influenza periods according to national influenza surveillance data. If more than one off-season estimate was provided, we decided to use the post-influenza season estimate for analysis (for a detailed description of the definition of “off-season” in the studies, see Additional file 4).

**Fig. 3:**
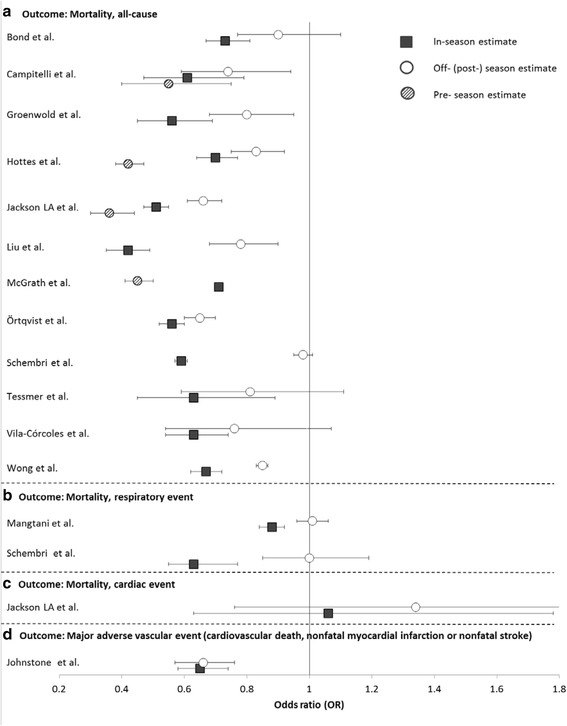
Odds ratios (95 % CIs) of influenza vaccine effectiveness during influenza seasons (black square), during pre-influenza seasons (striped circle) and post-influenza seasons (white circle) against all-cause mortality (**a**), death due to respiratory event (**b**), death due to cardiac event (**c**), and major adverse vascular event (**d**)

**Fig. 4:**
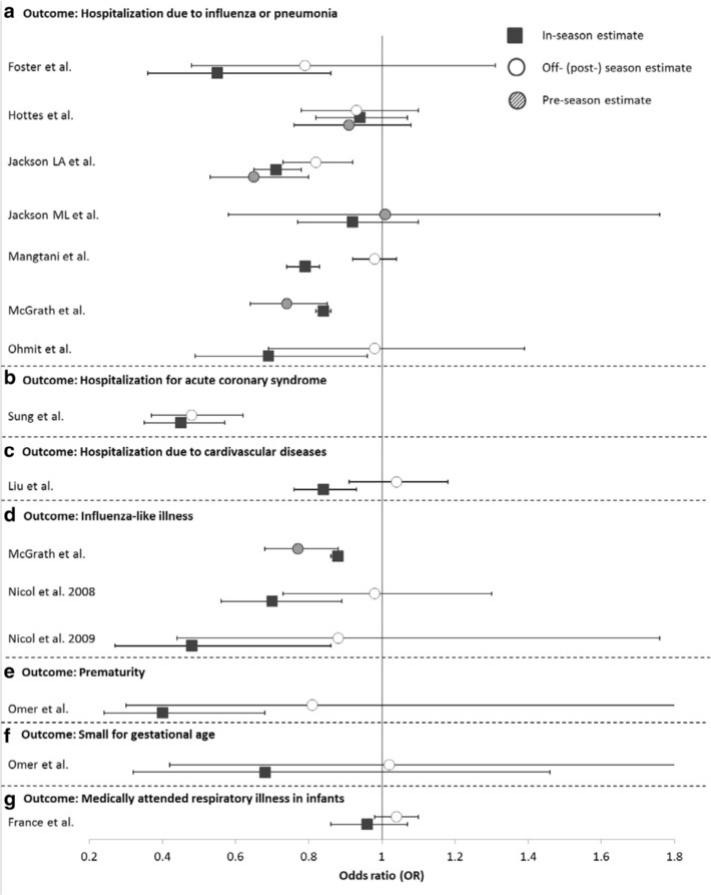
Odds ratios (95 % CIs) of influenza vaccine effectiveness during influenza seasons (black square), during pre-influenza seasons (striped circle) and post-influenza seasons (white circle) against hospitalization due to influenza or pneumonia (**a**), hospitalization for acute coronary syndrome (**b**), hospitalization due to cardiovascular diseases (**c**), influenza-like illness (**d**), prematurity (**e**), small for gestational age (**f**), and medically attended respiratory illness in infants (**g**)

Analyzing the 31 adjusted off-season estimates that were reported by the 23 included studies, we found statistically significant effects of influenza vaccination outside the influenza season in 13 studies (Figs. 3 and 4). Nine (39 %) of the 23 included studies reported at least one statistically significant VE estimate outside the influenza season (Figs. 3 and 4). These off-season effects were not restricted to the outcome all-cause mortality, but were also reported for four other outcomes (major adverse vascular events, hospitalization due to influenza/pneumonia, acute coronary syndrome, ILI). However, significant off-season estimates were more likely to occur when all-cause mortality was used as an outcome (8/13; 67 %) compared to other outcomes (5/19; 26 %; *p* = 0.03 by chi^2^ test). We then evaluated whether the occurrence of significant off-season estimates was related to the risk of healthy vaccinee bias, as judged from the baseline data of the respective study populations. We found that 46 % (6/13) of the significant off-season estimates were associated with high risk of healthy vaccinee bias at baseline. In contrast, only 6 % (1/18) of non-significant off-season estimates were associated with high risk of healthy vaccinee bias (*p* = 0.01 by chi^2^ test). Studies covering non-elderly populations did not report statistically significant off-season estimates for neither outcome.

## Discussion

In this review, we systematically assessed the frequency and impact of two major forms of bias/confounding commonly found in observational studies assessing influenza vaccine effectiveness. Our analysis revealed that the majority of included studies showed evidence for confounding by indication, as judged from the baseline characteristics of vaccinated and unvaccinated study participants. Analysis of crude and adjusted estimates showed that statistical adjustment for confounders corrected for this form of bias, at least partially. However, despite adjustment, nearly half of the studies still showed significant estimates of vaccine effectiveness outside the influenza season, which indicates the presence of unmeasured confounding due to healthy vaccinee bias. Remarkably, significant off-season estimates were not only observed in studies on all-cause mortality, but also regarding other outcomes. However, all outcomes that were used in the included studies were only based on clinical criteria, none of the studies used outcomes with laboratory confirmation of the virus.

At population level, implausibly high mortality benefits of influenza vaccination have been observed particularly in elderly persons. Observational studies found a reduction of mortality of about 50 %, while it was estimated that influenza-related mortality attributed to less than 10 % in this age-group [2]. These and other observations led to the hypothesis of healthy vaccinee bias [36]. In healthy vaccinee bias, healthy persons are preferentially vaccinated against influenza, while persons with comorbidities have a lower likelihood to get vaccinated. A small subset of unvaccinated frail and terminally ill patients are suggested to explain the large/implausible results regarding mortality mentioned above. Adjustment for conventional comorbidities as confounders has been suggested to insufficiently capture the functional status of this subgroup [35]. In fact, in the majority of included studies comorbidities were identified through ICD-codes in administrative databases, which have been shown to fail to control adequately for confounding [37].

On the contrary, other authors have suggested the opposite form of bias/confounding to be present in observational studies on influenza vaccination. They concluded that patients with comorbidities are preferentially vaccinated against influenza, which reflects current recommendations by the World Health Organization (WHO) and several National Immunization Technical Advisory Groups (NITAGs), but might result in confounding by indication [6, 38]. Looking at the baseline characteristics of the included studies, we found that the majority of studies showed indication for this type of confounding rather than for healthy vaccinee bias. Remarkably, our meta-analytic approach showed that adjustment for comorbidities had only a small impact on the point estimate of VE. Although this procedure increased the VE estimates in the majority of studies, which is consistent with removal of confounding by indication, the effect size changed on average by only 7 to 12 %. However, since nearly all studies adjusted for comorbidities and other confounders such as sex and age simultaneously in one single step, it is unclear whether and to what extent this effect can be attributed to removal of confounding by comorbidities.

Interestingly, the analyses performed in the study by Campitelli et al. [7] showed that it is possible to adjust, at least in part, for both forms of bias/confounding, given that enough information have been collected regarding comorbidities and functional status of study participants. Those authors demonstrated that the addition of comorbidities as confounders to the regression model shifted the effect estimate away from 1.0, which indicates correction for confounding by indication. They then added indicators of functional status to the model and observed a shift of the estimate towards 1.0, indicating correction for healthy vaccinee bias. However, additional analyses performed in this study demonstrated that residual confounding was likely to be still present in those data since adjustment for comorbidities and frailty indicators could not eliminate significant off-season estimates.

Our systematic review shows that these findings can be generalized to the body of literature on this issue. In nearly half of the studies identified here, significant off-season estimates were observed despite adjustment for confounders. Although significant off-season estimates were more likely to occur in studies which showed high risk of healthy vaccinee bias at baseline, they were also observed in studies that did not find indication of healthy vaccinee bias by comparing the characteristics of vaccinated and unvaccinated study participants. Interestingly, in studies covering non-elderly participants’ significant off-season estimates were not identified. However, there were only five of these studies and it is unclear whether this could be attributed to a lower prevalence of comorbidities or frailty indicators in these age groups or whether this is a chance finding.

The significance of healthy vaccinee bias as well as the suitability of off-season estimates as indicators for its presence has been debated in several publications. Nichol et al. discussed that influenza vaccination is common in patients with functional impairments and frailty, [6] speaking against the assumption that a terminally ill and frail subgroup of patients is responsible for the observation of off-season estimates. Hak et al. suggested that circulation of influenza in the few months before and after the influenza season might account for “off-season” estimates, as well as a prolonged impact of influenza on mortality which extends several months beyond illness [4]. On the contrary, the publication by Wong et al. [32] provided additional evidence that off-season estimates result from healthy vaccinee bias for which the conventional analysis failed to adjust for. Those authors used the same data base that was primarily analyzed as a cohort study to apply instrumental variable technique. Using this study design, they were able to show that quasi-randomization eliminates off-season effects of influenza vaccination, supporting the interpretation that study design and data analysis are crucial here.

The recent debate on bias in influenza VE studies mainly focusses on the outcome all-cause mortality [2, 6, 10, 35]. Our systematic review demonstrates that significant off-season estimates were also observed in the context of three other clinical outcomes, although significantly less frequent than in mortality studies. All of these outcomes have in common that they are based on unspecific case definitions without laboratory confirmation of influenza infection, which is likely to lead to outcome misclassification. It should be evaluated in future studies whether significant off-season VE estimates are still present when influenza-specific outcomes with laboratory confirmation are assessed.

Our study has several strengths. It is the first systematic review which focused on this issue and examined all published studies with relevant data for the assessment of these two types of bias. In addition, we quantified the extent to which adjustment could correct for confounding by indication regarding different clinical outcomes. However, some limitations of our study have to be addressed although they are mainly caused by limitations of the included studies: First, a number of studies could not be included since they did not provide enough information to assess risk of bias. Some of them included also more specific endpoints with laboratory confirmation. For this reason, the proportion of studies with such biases might be an overestimation. Second, as it is often the case in administrative database-related studies, multiple groups of authors used the same data base and potential overlap between study populations cannot be completely excluded. We detected for example potential overlap between the studies by Campitelli et al. [7] and Wong et al. [32]. Third, since studies used different covariates for confounder-adjusted VE estimates and different definition of influenza and off-season periods, direct comparison of the results have to be taken with caution. Furthermore, in nearly all studies statistical adjustment was made in multivariate analysis for a variety of confounder simultaneously. Those sets of confounders did not only include comorbidities, but also age, sex and demographic characteristics. Therefore, the adjusted odds ratios used for our analysis do not accurately reflect confounding by indication. Finally, other types of bias, such as errors in diagnosis or vaccination status, could also have influenced study findings but were not in the focus of our analysis.

## Conclusions

To conclude, this systematic review supports the hypothesis that confounding by indication and healthy vaccine bias operate simultaneously in observational studies on influenza vaccination using unspecific outcomes. Consequently, it seems impossible to infer whether the adjusted vaccine effectiveness estimates under- or overestimate the true effect of the vaccine. Cohort study designs using administrative data bases with unspecific outcomes such as all-cause mortality should no longer be used to measure the effects of influenza vaccination. Instead, other study designs, including test-negative design studies [39] and quasi-randomized studies using influenza-specific laboratory-confirmed outcomes, are needed to obtain more reliable estimates of influenza vaccine effectiveness. However, one should be aware that in these study types other forms of bias might operate. This should be assessed in further methodological studies.

## Additional files

Additional file 1:
**Protocol for systematic review: Frequency and impact of selection bias in observational studies assessing influenza vaccine effectiveness: A systematic review.** (DOCX 24 kb) Additional file 2:
**Search strategy for the systematic review on frequency and impact of selection bias in observational studies on influenza vaccine effectiveness.** (DOCX 21 kb) Additional file 3:
**List of excluded studies.** (DOCX 147 kb) Additional file 4:
**Definitions of influenza season periods and control periods (off-season) in the included studies.** (DOCX 28 kb)

## References

[1] Jefferson T, Di Pietrantonj C, Al-Ansary LA, Ferroni E, Thorning S, Thomas RE. Vaccines for preventing influenza in the elderly. Cochrane Database Syst Rev. 2010:CD004876. doi:10.1002/14651858. 10.1002/14651858.CD004876.pub3 20166072

[2] Simonsen L, Taylor RJ, Viboud C, Miller MA, Jackson LA (2007). Mortality benefits of influenza vaccination in elderly people: an ongoing controversy. Lancet Infect Dis.

[3] Jackson LA, Jackson ML, Nelson JC, Neuzil KM, Weiss NS (2006). Evidence of bias in estimates of influenza vaccine effectiveness in seniors. Int J Epidemiol.

[4] Hak E, Hoes AW, Nordin J, Nichol KL (2006). Benefits of influenza vaccine in US elderly--appreciating issues of confounding bias and precision. Int J Epidemiol.

[5] Jackson ML, Nelson JC, Weiss NS, Neuzil KM, Barlow W, Jackson LA (2008). Influenza vaccination and risk of community-acquired pneumonia in immunocompetent elderly people: a population-based, nested case–control study. Lancet.

[6] Nichol KL (2009). Challenges in evaluating influenza vaccine effectiveness and the mortality benefits controversy. Vaccine.

[7] Campitelli MA, Rosella LC, Stukel TA, Kwong JC (2010). Influenza vaccination and all-cause mortality in community-dwelling elderly in Ontario, Canada, a cohort study. Vaccine.

[8] Jackson ML, Yu O, Nelson JC, Naleway A, Belongia EA, Baxter R, Narwaney K, Jacobsen SJ, Shay DK, Jackson LA (2013). Further evidence for bias in observational studies of influenza vaccine effectiveness: the 2009 influenza A(H1N1) pandemic. Am J Epidemiol.

[9] Darvishian M, Gefenaite G, Turner RM, Pechlivanoglou P, Van der Hoek W, Van den Heuvel ER, Hak E (2014). After adjusting for bias in meta-analysis seasonal influenza vaccine remains effective in community-dwelling elderly. J Clin Epidemiol.

[10] Nelson JC, Jackson ML, Weiss NS, Jackson LA (2009). New strategies are needed to improve the accuracy of influenza vaccine effectiveness estimates among seniors. J Clin Epidemiol.

[11] Jackson ML, Weiss NS, Nelson JC, Jackson LA (2007). To rule out confounding, observational studies of influenza vaccine need to include analyses during the “preinfluenza period”. Arch Intern Med.

[12] Moher D, Liberati A, Tetzlaff J, Altman DG, Group P (2009). Preferred reporting items for systematic reviews and meta-analyses: the PRISMA statement. Ann Intern Med.

[13] Hrobjartsson A, Thomsen AS, Emanuelsson F, Tendal B, Hilden J, Boutron I, Ravaud P, Brorson S (2012). Observer bias in randomised clinical trials with binary outcomes: systematic review of trials with both blinded and non-blinded outcome assessors. BMJ.

[14] Hrobjartsson A, Thomsen AS, Emanuelsson F, Tendal B, Rasmussen JV, Hilden J, Boutron I, Ravaud P, Brorson S (2014). Observer bias in randomized clinical trials with time-to-event outcomes: systematic review of trials with both blinded and non-blinded outcome assessors. Int J Epidemiol.

[15] Ortqvist A, Granath F, Askling J, Hedlund J (2007). Influenza vaccination and mortality: prospective cohort study of the elderly in a large geographical area. Eur Respir J.

[16] Mangtani P, Cumberland P, Hodgson CR, Roberts JA, Cutts FT, Hall AJ (2004). A cohort study of the effectiveness of influenza vaccine in older people, performed using the United Kingdom general practice research database. J Infect Dis.

[17] Bond TC, Spaulding AC, Krisher J, McClellan W (2012). Mortality of dialysis patients according to influenza and pneumococcal vaccination status. Am J Kidney Dis.

[18] Foster DA, Talsma A, Furumoto-Dawson A, Ohmit SE, Margulies JR, Arden NH, Monto AS, Foster DA, Talsma A, Furumoto-Dawson A (1992). Influenza vaccine effectiveness in preventing hospitalization for pneumonia in the elderly. Am J Epidemiol.

[19] Groenwold RH, Hoes AW, Hak E (2009). Impact of influenza vaccination on mortality risk among the elderly. Eur Respir J.

[20] Hottes TS, Skowronski DM, Hiebert B, Janjua NZ, Roos LL, Van Caeseele P, Law BJ, De Serres G (2011). Influenza vaccine effectiveness in the elderly based on administrative databases: change in immunization habit as a marker for bias. PLoS One.

[21] Jackson LA, Yu O, Heckbert SR, Psaty BM, Malais D, Barlow WE, Thompson WW, Vaccine Safety Datalink Study G (2002). Influenza vaccination is not associated with a reduction in the risk of recurrent coronary events. Am J Epidemiol.

[22] Liu IF, Huang CC, Chan WL, Huang PH, Chung CM, Lin SJ, Chen JW, Leu HB (2012). Effects of annual influenza vaccination on mortality and hospitalization in elderly patients with ischemic heart disease: a nationwide population-based study. Prev Med.

[23] McGrath LJ, Kshirsagar AV, Cole SR, Wang L, Weber DJ, Sturmer T, Brookhart MA (2012). Influenza vaccine effectiveness in patients on hemodialysis: an analysis of a natural experiment. Arch Intern Med.

[24] Nichol KL, D’Heilly S, Ehlinger EP (2008). Influenza vaccination among college and university students: impact on influenzalike illness, health care use, and impaired school performance. Arch Pediatr Adolesc Med.

[25] Nichol KL, D’Heilly SJ, Greenberg ME, Ehlinger E (2009). Burden of influenza-like illness and effectiveness of influenza vaccination among working adults aged 50–64 years. Clin Infect Dis.

[26] Ohmit SE, Monto AS (1995). Influenza vaccine effectiveness in preventing hospitalization among the elderly during influenza type A and type B seasons. Int J Epidemiol.

[27] Omer SB, Goodman D, Steinhoff MC, Rochat R, Klugman KP, Stoll BJ, Ramakrishnan U (2011). Maternal influenza immunization and reduced likelihood of prematurity and small for gestational age births: a retrospective cohort study. PLoS Med.

[28] Schembri S, Morant S, Winter JH, MacDonald TM (2009). Influenza but not pneumococcal vaccination protects against all-cause mortality in patients with COPD. Thorax.

[29] Sung LC, Chen CI, Fang YA, Lai CH, Hsu YP, Cheng TH, Miser JS, Liu JC (2014). Influenza vaccination reduces hospitalization for acute coronary syndrome in elderly patients with chronic obstructive pulmonary disease: a population-based cohort study. Vaccine.

[30] Tessmer A, Welte T, Schmidt-Ott R, Eberle S, Barten G, Suttorp N, Schaberg T, Group Cs (2011). Influenza vaccination is associated with reduced severity of community-acquired pneumonia. Eur Respir J.

[31] Vila-Corcoles A, Rodriguez T, de Diego C, Ochoa O, Valdivieso A, Salsench E, Ansa X, Badia W, Saun N, Group ES (2007). Effect of influenza vaccine status on winter mortality in Spanish community-dwelling elderly people during 2002–2005 influenza periods. Vaccine.

[32] Wong K, Campitelli MA, Stukel TA, Kwong JC (2012). Estimating influenza vaccine effectiveness in community-dwelling elderly patients using the instrumental variable analysis method. Arch Intern Med.

[33] Johnstone J, Loeb M, Teo KK, Gao P, Dyal L, Liu L, Avezum A, Cardona-Munoz E, Sleight P, Fagard R (2012). Influenza vaccination and major adverse vascular events in high-risk patients. Circulation.

[34] France EK, Smith-Ray R, McClure D, Hambidge S, Xu S, Yamasaki K, Shay D, Weintraub E, Fry AM, Black SB (2006). Impact of maternal influenza vaccination during pregnancy on the incidence of acute respiratory illness visits among infants. Arch Pediatr Adolesc Med.

[35] Jackson LA, Nelson JC, Benson P, Neuzil KM, Reid RJ, Psaty BM, Heckbert SR, Larson EB, Weiss NS (2006). Functional status is a confounder of the association of influenza vaccine and risk of all cause mortality in seniors. Int J Epidemiol.

[36] Simonsen L, Viboud C, Taylor RJ, Miller MA, Jackson L (2009). Influenza vaccination and mortality benefits: new insights, new opportunities. Vaccine.

[37] Jackson ML, Nelson JC, Jackson LA (2011). Why do covariates defined by International Classification of Diseases codes fail to remove confounding in pharmacoepidemiologic studies among seniors?. Pharmacoepidemiol Drug Saf.

[38] Hak E, Verheij TJ, Grobbee DE, Nichol KL, Hoes AW (2002). Confounding by indication in non-experimental evaluation of vaccine effectiveness: the example of prevention of influenza complications. J Epidemiol Community Health.

[39] Jackson ML, Nelson JC (2013). The test-negative design for estimating influenza vaccine effectiveness. Vaccine.

