# Supplementary material for: Frequency and impact of confounding by indication and healthy vaccinee bias in observational studies assessing influenza vaccine effectiveness: a systematic review
Source: BMC Infect Dis. 2015 Oct 17;15:429. doi: 10.1186/s12879-015-1154-y (PMC4609091; doi:10.1186/s12879-015-1154-y)
Supplement: Additional file 1: — Protocol for systematic review: Frequency and impact of selection bias in observational studies assessing influenza vaccine effectiveness: A systematic review. (DOCX 24 kb) [file 12879_2015_1154_MOESM1_ESM.docx]

**Additional File 1**

Protocol for systematic review: Frequency and impact of selection bias in observational studies assessing influenza vaccine effectiveness: A systematic review

Cornelius Remschmidt, Thomas Harder, Ole Wichmann

1) Review questions:

a. How often do observational studies on influenza VE show indication of selection bias?

b. What is the impact of selection bias on VE point estimates?

c. How many of these studies show indication of residual confounding in the adjusted analyses?

According to the literature, the following factors might indicate selection bias in influenza VE studies:

(i) High risk of confounding by indication: In the baseline characteristics, vaccinated participants have a higher proportion of comorbidities compared to unvaccinated participants or inclusion of comorbidities in the regression model moves effectiveness estimate away from 1.0.

(ii) High risk of healthy vaccinee bias: Vaccinated participants have a lower proportion of comorbidities, as indicated by baseline characteristics or inclusion of comorbidities in the regression model moves effectiveness estimate towards 1.0 or significant effects of influenza vaccination appear outside the influenza season (“off-season estimates”), despite adjustment for comorbidities.

2) Data sources:

a. Electronic data bases: Medline, Embase, Cochrane Central Register of Controlled Trials, ClinicalTrials.gov. Databases are searched via the German Institute of Medical Documentation and Information (DIMDI) surface (text field search; available at: http://www.dimdi.de/static/en/index.html):

b. Reference lists of all identified studies

c. Restrictions: none regarding language or publication type

3) Search strategy: The following search strategy will be applied, using the text field search option via DIMDI:

#1 “influenza”

#2 “vacci*”

#3 “off-season”

#4 “summer”

#5 “control period”

#6 “bias”

#7 “observational”

#8 “case-control”

#9 “cohort”

#10 “retrospective”

#11 #1 AND #2

#12 #3 OR #4 OR #5 OR #6 OR #7 OR #8 OR #9 OR #10

#13 #11 AND #12

(restrictions: species: human)

4) Population/participants: Study participants of all ages with and without chronic diseases.

5) Intervention: Immunization with an approved vaccine against seasonal influenza

6) Comparator: Placebo or no vaccination

7) Study designs: Observational (non-randomized) studies

8) Outcomes: Studies will be included if they fulfill the following criteria: (i) baseline characteristics of vaccinated and unvaccinated participants reported; (ii) data on at least one clinical outcome is reported; (iii) crude and confounder-adjusted VE estimates from at least one influenza season and confounder-adjusted VE estimates from at least one “control” period outside the influenza season are reported

9) Data extraction: Two independent reviewers will screen studies by title and abstract for eligibility. Potential disagreements will be solved by discussion or by involving the third reviewer (OW). Identified studies will be retrieved in full text. The following data will be extracted: country, study design, age, sex, characteristics of study population (e.g., patients with underlying comorbidities), source of patient data, identification of clinical outcomes and vaccination status, definition of influenza season and off-season, and population size. In addition, we extracted data on crude and adjusted VE point estimates for all reported outcomes during influenza seasons, adjusted off-season point estimates, and which confounder were considered. Extraction forms will be pilot tested with the first two identified studies.

10) Risk of bias assessment: We will use the predefined criteria for confounding by indication or healthy vaccine bias, respectively.

11) Data synthesis: Data will be aggregated in tables, showing study characteristics and in- and off-season effect estimates. We will compare crude VE estimates to confounder-adjusted (fully-adjusted model) in-season estimates from the same study. In addition, we will quantify the extent by which adjustment for confounders move the in-season estimate away from the crude estimate for each outcome (Hrobjartsson et al, 2012 and 2014). To assess the impact of adjustment for confounders, we will meta-analyze the ratios of odds ratios for each outcome separately (random-effects models with inverse-variance methods).
