# Supplementary material for: Frequency and impact of confounding by indication and healthy vaccinee bias in observational studies assessing influenza vaccine effectiveness: a systematic review
Source: BMC Infect Dis. 2015 Oct 17;15:429. doi: 10.1186/s12879-015-1154-y (PMC4609091; doi:10.1186/s12879-015-1154-y)
Supplement: Additional file 2: — Search strategy for the systematic review on frequency and impact of selection bias in observational studies on influenza vaccine effectiveness. (DOCX 21 kb) [file 12879_2015_1154_MOESM2_ESM.docx]

**Additional File 2**

Search strategy for the systematic review on frequency and impact of selection bias in observational studies on influenza vaccine effectiveness.

#1 “influenza”

#2 “vacci*”

#3 “off-season”

#4 “summer”

#5 “control period”

#6 “bias”

#7 “observational”

#8 “case-control”

#9 “cohort”

#10 “retrospective”

#11 #1 AND #2

#12 #3 OR #4 OR #5 OR #6 OR #7 OR #8 OR #9 OR #10

#13 #11 AND #12

Databases searched via the German Institute of Medical Documentation and Information surface (text field search; available at: http://www.dimdi.de/static/en/index.html): MEDLINE, EMBASE and Cochrane Central Register of Controlled Trials from inception to May, 25, 2014; restrictions: species: human
