# Supplementary material for: Frequency and impact of confounding by indication and healthy vaccinee bias in observational studies assessing influenza vaccine effectiveness: a systematic review
Source: BMC Infect Dis. 2015 Oct 17;15:429. doi: 10.1186/s12879-015-1154-y (PMC4609091; doi:10.1186/s12879-015-1154-y)
Supplement: Additional file 3: — List of excluded studies. (DOCX 147 kb) [file 12879_2015_1154_MOESM3_ESM.docx]

**Additional File 3**

List of excluded studies (n=85):

No off-season estimates (n=43): [[1-43](#_ENREF_1)]

No relevant data (n=15): [[44-58](#_ENREF_44)]

No baseline data (n=12): [[59-70](#_ENREF_59)]

No unadjusted VE (n=8): [[71-78](#_ENREF_71)]

No unvaccinated control group (n=2): [[79](#_ENREF_79), [80](#_ENREF_80)]

Subgroup analyses of already included study (n=2): [[81](#_ENREF_81), [82](#_ENREF_82)]

Only off-season estimates (n=1): [[83](#_ENREF_83)]

Case series (n=1): [[84](#_ENREF_84)]

Duplicate (n=1)

**Excluded studies:**

1. Armstrong BG, Mangtani P, Fletcher A, Kovats S, McMichael A, Pattenden S, Wilkinson P: **Effect of influenza vaccination on excess deaths occurring during periods of high circulation of influenza: cohort study in elderly people.** *BMJ* 2004, **329:**660.

2. Keitel WA, Cate TR, Couch RB, Huggins LL, Hess KR: **Efficacy of repeated annual immunization with inactivated influenza virus vaccines over a five year period.** *Vaccine* 1997, **15:**1114-1122.

3. Szilagyi PG, Fairbrother G, Griffin MR, Hornung RW, Donauer S, Morrow A, Altaye M, Zhu Y, Ambrose S, Edwards KM, et al: **Influenza vaccine effectiveness among children 6 to 59 months of age during 2 influenza seasons: a case-cohort study.** *Arch Pediatr Adolesc Med* 2008, **162:**943-951.

4. Fukushima W, Hayashi Y, Mizuno Y, Suzuki K, Kase T, Ohfuji S, Fujieda M, Maeda A, Hirota Y: **Selection bias in evaluating of influenza vaccine effectiveness: a lesson from an observational study of elderly nursing home residents.** *Vaccine* 2008, **26:**6466-6469.

5. Hara M, Sakamoto T, Tanaka K: **Influenza vaccine effectiveness among elderly persons living in the community during the 2003--2004 season.** *Vaccine* 2008, **26:**6477-6480.

6. Skull SA, Andrews RM, Byrnes GB, Kelly HA, Nolan TM, Brown GV, Campbell DA: **Prevention of community-acquired pneumonia among a cohort of hospitalized elderly: benefit due to influenza and pneumococcal vaccination not demonstrated.** *Vaccine* 2007, **25:**4631-4640.

7. Tsai YW, Huang WF, Wen YW, Chen PF: **The relationship between influenza vaccination and outpatient visits for upper respiratory infection by the elderly in Taiwan.** *Value Health* 2007, **10:**117-127.

8. Landi F, Onder G, Cesari M, Russo A, Barillaro C, Bernabei R, Group S-HS, Gambassi G, Manigrasso L, Pagano F, Gobbi C: **In a prospective observational study, influenza vaccination prevented hospitalization among older home care patients.** *J Clin Epidemiol* 2006, **59:**1072-1077.

9. Looijmans-Van den Akker I, Verheij TJ, Buskens E, Nichol KL, Rutten GE, Hak E: **Clinical effectiveness of first and repeat influenza vaccination in adult and elderly diabetic patients.** *Diabetes Care* 2006, **29:**1771-1776.

10. Hara M, Sakamoto T, Tanaka K: **Effectiveness of influenza vaccination in preventing influenza-like illness among community-dwelling elderly: population-based cohort study in Japan.** *Vaccine* 2006, **24:**5546-5551.

11. Jackson LA, Nelson JC, Benson P, Neuzil KM, Reid RJ, Psaty BM, Heckbert SR, Larson EB, Weiss NS: **Functional status is a confounder of the association of influenza vaccine and risk of all cause mortality in seniors.** *Int J Epidemiol* 2006, **35:**345-352.

12. Montes M, Vicente D, Perez-Yarza EG, Cilla G, Perez-Trallero E: **Influenza-related hospitalisations among children aged less than 5 years old in the Basque Country, Spain: a 3-year study (July 2001-June 2004).** *Vaccine* 2005, **23:**4302-4306.

13. Hak E, Buskens E, van Essen GA, de Bakker DH, Grobbee DE, Tacken MA, van Hout BA, Verheij TJ: **Clinical effectiveness of influenza vaccination in persons younger than 65 years with high-risk medical conditions: the PRISMA study.** *Arch Intern Med* 2005, **165:**274-280.

14. Landi F, Onder G, Cesari M, Gravina EM, Lattanzio F, Russo A, Bernabei R, Group S-HS: **Effects of influenza vaccination on mortality among frail, community-living elderly patients: an observational study.** *Aging Clin Exp Res* 2003, **15:**254-258.

15. Voordouw BC, van der Linden PD, Simonian S, van der Lei J, Sturkenboom MC, Stricker BH: **Influenza vaccination in community-dwelling elderly: impact on mortality and influenza-associated morbidity.** *Arch Intern Med* 2003, **163:**1089-1094.

16. Nichol KL, Nordin J, Mullooly J, Lask R, Fillbrandt K, Iwane M: **Influenza vaccination and reduction in hospitalizations for cardiac disease and stroke among the elderly.** *N Engl J Med* 2003, **348:**1322-1332.

17. Gutierrez EB, Li HY, Santos AC, Lopes MH: **Effectiveness of influenza vaccination in elderly outpatients in Sao Paulo city, Brazil.** *Rev Inst Med Trop Sao Paulo* 2001, **43:**317-320.

18. Monto AS, Hornbuckle K, Ohmit SE: **Influenza vaccine effectiveness among elderly nursing home residents: a cohort study.** *Am J Epidemiol* 2001, **154:**155-160.

19. Wood SC, Alexseiv A, Nguyen VH: **Effectiveness and economical impact of vaccination against influenza among a working population in Moscow.** *Vaccine* 1999, **17 Suppl 3:**S81-87.

20. Chan TC, Hung IF, Luk JK, Shea YF, Chan FH, Woo PC, Chu LW: **Efficacy of dual vaccination of pandemic H1N1 2009 influenza and seasonal influenza on institutionalized elderly: a one-year prospective cohort study.** *Vaccine* 2011, **29:**7773-7778.

21. Manzur A, Izquierdo C, Ruiz L, Sousa D, Bayas JM, Celorrio JM, Varona W, Nebot M, Salleras L, Dominguez A, et al: **Influence of prior pneumococcal and influenza vaccination on outcomes of older adults with community-acquired pneumonia.** *J Am Geriatr Soc* 2011, **59:**1711-1716.

22. Larrauri A, Savulescu C, Jimenez-Jorge S, Perez-Brena P, Pozo F, Casas I, Ledesma J, de Mateo S, Spanish Influenza Surveillance S: **Influenza pandemic (H1N1) 2009 activity during summer 2009. Effectiveness of the 2008-9 trivalent vaccine against pandemic influenza in Spain.** *Gac Sanit* 2011, **25:**23-28.

23. Hashim AB, McKeever T, Kelly SJ, Nguyen-Van-Tam JS: **Evaluation of inter-pandemic influenza vaccine effectiveness during eight consecutive winter seasons in England and Wales in patients with cardiovascular risk factors.** *J Infect Public Health* 2010, **3:**159-165.

24. Jick H, Hagberg KW: **Effectiveness of influenza vaccination in the United kingdom, 1996-2007.** *Pharmacotherapy* 2010, **30:**1199-1206.

25. Hung IF, Leung AY, Chu DW, Leung D, Cheung T, Chan CK, Lam CL, Liu SH, Chu CM, Ho PL, et al: **Prevention of acute myocardial infarction and stroke among elderly persons by dual pneumococcal and influenza vaccination: a prospective cohort study.** *Clin Infect Dis* 2010, **51:**1007-1016.

26. Langley JM, Dodds L, Fell D, Langley GR: **Pneumococcal and influenza immunization in asplenic persons: a retrospective population-based cohort study 1990-2002.** *BMC Infect Dis* 2010, **10:**219.

27. Manzoli L, Villari P, Granchelli C, Savino A, Carunchio C, Alessandrini M, Palumbo F, De Vito C, Schioppa F, Di Stanislao F, Boccia A: **Influenza vaccine effectiveness for the elderly: a cohort study involving general practitioners from Abruzzo, Italy.** *J Prev Med Hyg* 2009, **50:**109-112.

28. Lee WJ, Chen LK, Tang GJ, Lan TY: **The impact of influenza vaccination on hospitalizations and mortality among frail older people.** *J Am Med Dir Assoc* 2014, **15:**256-260.

29. Kopel E, Klempfner R, Goldenberg I: **Influenza vaccine and survival in acute heart failure.** *Eur J Heart Fail* 2014, **16:**264-270.

30. Debin M, Colizza V, Blanchon T, Hanslik T, Turbelin C, Falchi A: **Effectiveness of 2012-2013 influenza vaccine against influenza-like illness in general population: estimation in a French web-based cohort.** *Hum Vaccin Immunother* 2014, **10:**536-543.

31. Macintyre CR, Heywood AE, Kovoor P, Ridda I, Seale H, Tan T, Gao Z, Katelaris AL, Siu HW, Lo V, et al: **Ischaemic heart disease, influenza and influenza vaccination: a prospective case control study.** *Heart* 2013, **99:**1843-1848.

32. Huang CL, Nguyen PA, Kuo PL, Iqbal U, Hsu YH, Jian WS: **Influenza vaccination and reduction in risk of ischemic heart disease among chronic obstructive pulmonary elderly.** *Comput Methods Programs Biomed* 2013, **111:**507-511.

33. He Q, Xu J, Chen X, Lu J, Li K, Li Z, Wang M, Yang Q, Dong Z, Liu X, et al: **Effectiveness of seasonal influenza vaccine against clinically diagnosed influenza over 2 consecutive seasons in children in Guangzhou, China: a matched case-control study.** *Hum Vaccin Immunother* 2013, **9:**1720-1724.

34. Wang IK, Lin CL, Lin PC, Liang CC, Liu YL, Chang CT, Yen TH, Morisky DE, Huang CC, Sung FC: **Effectiveness of influenza vaccination in patients with end-stage renal disease receiving hemodialysis: a population-based study.** *PLoS One* 2013, **8:**e58317.

35. Mahamat A, Daures JP, de Wzieres B: **Additive preventive effect of influenza and pneumococcal vaccines in the elderly: results of a large cohort study.** *Hum Vaccin Immunother* 2013, **9:**128-135.

36. Chiu PJ, Chen CH, Chih YC: **Effectiveness of the influenza vaccination program for the elderly in Taiwan.** *Vaccine* 2013, **31:**632-638.

37. Wang IK, Lin CL, Chang YC, Lin PC, Liang CC, Liu YL, Chang CT, Yen TH, Huang CC, Sung FC: **Effectiveness of influenza vaccination in elderly diabetic patients: a retrospective cohort study.** *Vaccine* 2013, **31:**718-724.

38. Chan TC, Hung IF, Luk JK, Shea YF, Chan FH, Woo PC, Chu LW: **Functional status of older nursing home residents can affect the efficacy of influenza vaccination.** *J Gerontol A Biol Sci Med Sci* 2013, **68:**324-330.

39. Chang YC, Chou YJ, Liu JY, Yeh TF, Huang N: **Additive benefits of pneumococcal and influenza vaccines among elderly persons aged 75 years or older in Taiwan--a representative population-based comparative study.** *J Infect* 2012, **65:**231-238.

40. Yang P, Zhang L, Shi W, Lu G, Cui S, Peng X, Zhang D, Liu Y, Liang H, Pang X, Wang Q: **Seroprevalence of pandemic (H1N1) 2009 influenza and effectiveness of 2010/2011 influenza vaccine during 2010/2011 season in Beijing, China.** *Influenza Other Respir Viruses* 2012, **6:**381-388.

41. Chen CI, Sung LC, Miser JS, Fang YA, Tsai CQ, Liu JC, Li YC: **Influenza Vaccination May Lead to Reduction of Hospitalization for Heart Failure in Elderly Patients with Chronic Obstructive Pulmonary Disease.** *Journal of Experimental and Clinical Medicine* 2013, **5:**65-68.

42. Vamos EP, Pape U, Curcin V: **Influenza vaccine effectiveness against hospitalisation and death in people with Type 2 diabetes.** *Diabetic Medicine* 2014, **31:**28-183.

43. Hardelid P, Fleming DM, Andrews N, Barley M, Durnall H, Mangtani P, Pebody R: **Effectiveness of trivalent and pandemic influenza vaccines in England and Wales 2008-2010: results from a cohort study in general practice.** *Vaccine* 2012, **30:**1371-1378.

44. Mori M, Oura A, Ohnishi H, Washio M: **Confounding in evaluating the effectiveness of influenza vaccine.** *Vaccine* 2008, **26:**6459-6461.

45. Orenstein EW, De Serres G, Haber MJ, Shay DK, Bridges CB, Gargiullo P, Orenstein WA: **Methodologic issues regarding the use of three observational study designs to assess influenza vaccine effectiveness.** *Int J Epidemiol* 2007, **36:**623-631.

46. Yoo BK, Grosse S, Frick KD: **Self-selection and evaluation of influenza vaccination effectiveness among elderly.** *Vaccine* 2006, **24:**6374-6375.

47. Kawai S, Nanri S, Ban E, Inokuchi M, Tanaka T, Tokumura M, Kimura K, Sugaya N: **Influenza vaccination of schoolchildren and influenza outbreaks in a school.** *Clin Infect Dis* 2011, **53:**130-136.

48. Cohen SA, Chui KK, Naumova EN: **Influenza vaccination in young children reduces influenza-associated hospitalizations in older adults, 2002-2006.** *J Am Geriatr Soc* 2011, **59:**327-332.

49. Nelson JC, Jackson ML, Weiss NS, Jackson LA: **New strategies are needed to improve the accuracy of influenza vaccine effectiveness estimates among seniors.** *J Clin Epidemiol* 2009, **62:**687-694.

50. Rogawski E, McGrath L, Vielot N, Westreich D: **Ischaemic heart disease, influenza and influenza vaccination: a prospective case control study.** *Heart* 2014, **100:**517-518.

51. Lian Ie B, Wu HD, Chang WT, Chao DY: **The temporal trend of influenza-associated morbidity and the impact of early appearance of antigenic drifted strains in a Southeast Asian country.** *PLoS One* 2014, **9:**e84239.

52. Nelson JC, Marsh T, Lumley T, Larson EB, Jackson LA, Jackson ML, Vaccine Safety Datalink T: **Validation sampling can reduce bias in health care database studies: an illustration using influenza vaccination effectiveness.** *J Clin Epidemiol* 2013, **66:**S110-121.

53. Locher JL, Rucks AC, Spencer SA, Pettaway GJ, Kilgore ML: **Influenza immunization in older adults with and without cancer.** *J Am Geriatr Soc* 2012, **60:**2099-2103.

54. Crowcroft NS, Rosella LC: **The potential effect of temporary immunity as a result of bias associated with healthy users and social determinants on observations of influenza vaccine effectiveness; could unmeasured confounding explain observed links between seasonal influenza vaccine and pandemic H1N1 infection?** *BMC Public Health* 2012, **12:**458.

55. Schooling CM, McGhee SM, Cowling BJ, Thomas GN, Chan WM, Ho KS, Wong VC, Leung GM: **Influenza vaccination and hospitalisation in Elderly Health Centres.** *Hong Kong Med J* 2012, **18 Suppl 2:**4-7.

56. Loomba RS, Aggarwal S, Shah PH, Arora RR: **Influenza vaccination and cardiovascular morbidity and mortality: analysis of 292,383 patients.** *J Cardiovasc Pharmacol Ther* 2012, **17:**277-283.

57. Ferdinands JM, Shay DK: **Magnitude of potential biases in a simulated case-control study of the effectiveness of influenza vaccination.** *Clin Infect Dis* 2012, **54:**25-32.

58. Ho TY, Huang KY, Huang TT, Huang YS, Ho HC, Chou P, Lin CH, Wei CK, Lian WC, Chen TC, et al: **The impact of influenza vaccinations on the adverse effects and hospitalization rate in the elderly: a national based study in an Asian country.** *PLoS One* 2012, **7:**e50337.

59. Christenson B, Pauksen K, Sylvan SP: **Effect of influenza and pneumococcal vaccines in elderly persons in years of low influenza activity.** *Virol J* 2008, **5:**52.

60. Grau AJ, Fischer B, Barth C, Ling P, Lichy C, Buggle F: **Influenza vaccination is associated with a reduced risk of stroke.** *Stroke* 2005, **36:**1501-1506.

61. Voordouw AC, Sturkenboom MC, Dieleman JP, Stijnen T, Smith DJ, van der Lei J, Stricker BH: **Annual revaccination against influenza and mortality risk in community-dwelling elderly persons.** *JAMA* 2004, **292:**2089-2095.

62. Colquhoun AJ, Nicholson KG, Botha JL, Raymond NT: **Effectiveness of influenza vaccine in reducing hospital admissions in people with diabetes.** *Epidemiol Infect* 1997, **119:**335-341.

63. Baxter R, Lee J, Fireman B: **Evidence of bias in studies of influenza vaccine effectiveness in elderly patients.** *J Infect Dis* 2010, **201:**186-189.

64. Baxter R, Ray GT, Fireman BH: **Effect of influenza vaccination on hospitalizations in persons aged 50 years and older.** *Vaccine* 2010, **28:**7267-7272.

65. Fireman B, Lee J, Lewis N, Bembom O, van der Laan M, Baxter R: **Influenza vaccination and mortality: differentiating vaccine effects from bias.** *Am J Epidemiol* 2009, **170:**650-656.

66. Skull SA, Andrews RM, Byrnes GB, Campbell DA, Kelly HA, Brown GV, Nolan TM: **Hospitalized community-acquired pneumonia in the elderly: an Australian case-cohort study.** *Epidemiol Infect* 2009, **137:**194-202.

67. Siriwardena AN, Asghar Z, Coupland CC: **Influenza and pneumococcal vaccination and risk of stroke or transient ischaemic attack-matched case control study.** *Vaccine* 2014, **32:**1354-1361.

68. Ridenhour BJ, Campitelli MA, Kwong JC, Rosella LC, Armstrong BG, Mangtani P, Calzavara AJ, Shay DK: **Effectiveness of inactivated influenza vaccines in preventing influenza-associated deaths and hospitalizations among Ontario residents aged >/= 65 years: estimates with generalized linear models accounting for healthy vaccinee effects.** *PLoS One* 2013, **8:**e76318.

69. Fu C, He Q, Li Z, Xu J, Li Y, Lu J, Li K, Yang Q, Dong Z, Liu X, et al: **Seasonal influenza vaccine effectiveness among children, 2010-2012.** *Influenza Other Respir Viruses* 2013, **7:**1168-1174.

70. Puig-Barbera J, Diez-Domingo J, Perez Hoyos S, Belenguer Varea A, Gonzalez Vidal D: **Effectiveness of the MF59-adjuvanted influenza vaccine in preventing emergency admissions for pneumonia in the elderly over 64 years of age.** *Vaccine* 2004, **23:**283-289.

71. Nichol KL, Nordin JD, Nelson DB, Mullooly JP, Hak E: **Effectiveness of influenza vaccine in the community-dwelling elderly.** *N Engl J Med* 2007, **357:**1373-1381.

72. Heffelfinger JD, Heckbert SR, Psaty BM, Weiss NS, Thompson WW, Bridges CB, Jackson LA: **Influenza vaccination and risk of incident myocardial infarction.** *Hum Vaccin* 2006, **2:**161-166.

73. Voordouw BC, Sturkenboom MC, Dieleman JP, Stijnen T, van der Lei J, Stricker BH: **Annual influenza vaccination in community-dwelling elderly individuals and the risk of lower respiratory tract infections or pneumonia.** *Arch Intern Med* 2006, **166:**1980-1985.

74. Heymann AD, Shapiro Y, Chodick G, Shalev V, Kokia E, Kramer E, Shemer J: **Reduced hospitalizations and death associated with influenza vaccination among patients with and without diabetes.** *Diabetes Care* 2004, **27:**2581-2584.

75. Nichol KL, Baken L, Nelson A: **Relation between influenza vaccination and outpatient visits, hospitalization, and mortality in elderly persons with chronic lung disease.** *Ann Intern Med* 1999, **130:**397-403.

76. Patrick AR, Brookhart MA: **Confounding in Studies of Influenza Vaccination and Mortality in the Elderly.** *Pharmacoepidemiology and Drug Safety* 2010, **19:**S1-S347.

77. Jackson ML, Yu O, Nelson JC, Naleway A, Belongia EA, Baxter R, Narwaney K, Jacobsen SJ, Shay DK, Jackson LA: **Further evidence for bias in observational studies of influenza vaccine effectiveness: the 2009 influenza A(H1N1) pandemic.** *American journal of epidemiology* 2013, **178:**1327-1336.

78. Lau D, Eurich DT, Majumdar SR, Katz A, Johnson JA: **Effectiveness of influenza vaccination in working-age adults with diabetes: a population-based cohort study.** *Thorax* 2013, **68:**658-663.

79. Puig-Barbera J, Natividad-Sancho A, Calabuig-Perez J, Lluch-Rodrigo JA, Pastor-Villalba E, Martinez-Ubeda S, Perez-Vilar S, Diez-Domingo J: **MF59-adjuvanted and virosomal influenza vaccines for preventing influenza hospitalization in older people: comparative effectiveness using the Valencia health care information system.** *Vaccine* 2013, **31:**3995-4002.

80. Mannino S, Villa M, Apolone G, Weiss NS, Groth N, Aquino I, Boldori L, Caramaschi F, Gattinoni A, Malchiodi G, Rothman KJ: **Effectiveness of adjuvanted influenza vaccination in elderly subjects in northern Italy.** *Am J Epidemiol* 2012, **176:**527-533.

81. de Diego C, Vila-Corcoles A, Ochoa O, Rodriguez-Blanco T, Salsench E, Hospital I, Bejarano F, Del Puy Muniain M, Fortin M, Canals M, Group ES: **Effects of annual influenza vaccination on winter mortality in elderly people with chronic heart disease.** *Eur Heart J* 2009, **30:**209-216.

82. Rodriguez-Blanco T, Vila-Corcoles A, de Diego C, Ochoa-Gondar O, Valdivieso E, Bobe F, Morro A, Hernandez N, Martin A, Calamote F, et al: **Relationship between annual influenza vaccination and winter mortality in diabetic people over 65 years.** *Hum Vaccin Immunother* 2012, **8:**363-370.

83. Eurich DT, Marrie TJ, Johnstone J, Majumdar SR: **Mortality reduction with influenza vaccine in patients with pneumonia outside "flu" season: pleiotropic benefits or residual confounding?** *Am J Respir Crit Care Med* 2008, **178:**527-533.

84. Gwini SM, Coupland CA, Siriwardena AN: **The effect of influenza vaccination on risk of acute myocardial infarction: self-controlled case-series study.** *Vaccine* 2011, **29:**1145-1149.
