# Supplementary material for: Frequency and impact of confounding by indication and healthy vaccinee bias in observational studies assessing influenza vaccine effectiveness: a systematic review
Source: BMC Infect Dis. 2015 Oct 17;15:429. doi: 10.1186/s12879-015-1154-y (PMC4609091; doi:10.1186/s12879-015-1154-y)
Supplement: Additional file 4: — Definitions of influenza season periods and control periods (off-season) in the included studies. (DOCX 28 kb) [file 12879_2015_1154_MOESM4_ESM.docx]

**Additional File 4**

Definitions of influenza season periods and control periods (off-season) in the included studies.

| Author, year | Method/ source for definition  of influenza season | Definition of influenza season period | Definition of off-season period(s) |
| --- | --- | --- | --- |
| Bond, 2012 | Not explicitly defined | Not explicitly defined | VE calculated by 3 months spans (i.e., January-March, February-April, March-May, etc.) |
| Campitelli, 2010 | National influenza surveillance data | First/last occurrences of two consecutive weeks with ≥ 5% of specimen tested positive for influenza | Pre-season: September, 1 to start season Post-season: end season to August 31 |
| France, 2006 | National influenza surveillance data | Peak season: ≥ 5% of specimen tested positive for influenza | Summer period: July 1 to September 30 |
| Foster, 1992 | Community-based sentinel network | Peak season: ≥ 5% of specimen tested positive for influenza | Low or absent circulation: < 5% positive for influenza, i.e. November, March and April |
| Groenwold, 2009 | Influenza surveillance data | ≥ 2 consecutive weeks in which each week accounted for at least 5% of the season’s total number of influenza isolates | Summer period: week 20 to week 40 of each year |
| Hottes, 2011 | Weekly counts of influenza isolates recorded by Cadham Provincial Laboratory | First and last occurrences of at least two consecutive weeks with two or more influenza isolates reported | 6 different periods defined: fall, pre-influenza, influenza, peak, spring and summer (week 24-week 34) |
| Jackson LA, 2006 | National influenza surveillance data | First and last weeks with at least 50 influenza isolates reported. | Pre-season: September, 1 to start season Post-season: end season to August 31 |
| Jackson LA, 2002 | Not reported | Influenza circulation period: November-April | Influenza noncirculation period: May-October |
| Jackson ML, 2008 | National influenza surveillance data | First and last weeks with at least 50 influenza isolates reported. | Pre-season: time from availability of influenza vaccine to start of influenza season |
| Johnstone, 2012 | World Health Organization Global Influenza Surveillance Network | Southern hemisphere: June-November and as December-May for the northern hemisphere. | Off-season: All weeks not included in the influenza season for a particular study year |
| Liu, 2012 | Taiwan Center of Disease Control | October-March | April-September |
| Mangtani, 2004 | National GP surveillance system | GP-consultation rates for ILI ≥ 50/ 100,000 person-weeks | Periinfluenza-season: weeks between November 15 to April 30 outside influenza season Summer season: May-August |
| McGrath, 2012 | National influenza surveillance data | First week during which > 10% of isolates positive for influenza ^1^ | Preinfluenza seasons: September 1 to begin of influenza season |
| Nicol, 2008 | Influenza surveillance data | 2002/03 and 05/06: January-March 2003/04: December-February 2004/05: December-March | Noninfluenza period: months between November and April outside influenza seasons |
| Nicol, 2009 | Influenza surveillance data | Months for which influenza activity was local, regional, or widespread | Noninfluenza period: months between November and April outside influenza season |
| Ohmit, 1995 | Community-based sentinel network | Peak season: ≥ 5% of specimen tested positive for influenza | Low/absent circulation: < 5% positive for influenza |
| Omer, 2011 | Influenza surveillance data | Influenza season divided into period of local, regional and widespread influenza activity ^2^ | Pre-influenza period: time between October and begin of local influenza activity |
| Örtqvist, 2007 | National influenza surveillance data | December-April | Off-season: May-August |
| Schembri, 2009 | Not reported | December-March | Off-season: April-November |
| Sung, 2014 | Taiwan Center of Disease Control | October-March | Non-influenza season: April-September |
| Tessmer, 2011 | National influenza surveillance data | December-April | Off-season: May-November |
| Vila-Corcoles, 2007 | National influenza surveillance data | January-April | Control period: July-August |
| Wong, 2012 | Laboratory-based sentinel network | First/last occurrences of two consecutive weeks with ≥ 5% of specimen tested positive for influenza | Post–influenza seasons: July-September after each influenza season |
| GP, general practitioner; ILI, influenza-like illness ^1^ Sensitivity analysis additionally reported with influenza seasons defined as the week with 5% of isolates positive for influenza; ^2^ point estimates during the period of local influenza activity (that also included periods of regional and widespread activity) were used | | | |
